# Micro Versus Macro – The Effect of Environmental Confinement on Cellular Nanoparticle Uptake

**DOI:** 10.3389/fbioe.2020.00869

**Published:** 2020-07-24

**Authors:** Viraj G. Damle, Rokshana Sharmin, Aryan Morita, Linyan Nie, Romana Schirhagl

**Affiliations:** ^1^Department of Biomedical Engineering, University Medical Center Groningen, University of Groningen, Groningen, Netherlands; ^2^Department of Dental Biomedical Sciences, Faculty of Dentistry, Universitas Gadjah Mada, Yogyakarta, Indonesia

**Keywords:** microfluidic cell cultures, nanoparticles, fluorescent nanodiamonds, macrophages, cellular uptake

## Abstract

While the microenvironment is known to alter the cellular behavior in terms of metabolism, growth and the degree of endoplasmic reticulum stress, its influence on the nanoparticle uptake is not yet investigated. Specifically, it is not clear if the cells cultured in a microenvironment ingest different amounts of nanoparticles than cells cultured in a macroenvironment (for example a petri dish). To answer this question, here we used J774 murine macrophages and fluorescent nanodiamonds (FND) as a model system to systematically compare the uptake efficiency of cells cultured in a petri dish and in a microfluidic channel. Specifically, equal numbers of cells were cultured in two devices followed by the FND incubation. Then cells were fixed, stained and imaged to quantify the FND uptake. We show that the FND uptake in the cells cultured in petri dishes is significantly higher than the uptake in a microfluidic chip where the alteration in CO_2_ environment, the cell culture medium pH and the surface area to volume ratio seem to be the underlying causes leading to this observed difference.

## Introduction

Microfluidic technology is highly sought after in the field of therapeutics (Dittrich and Manz, 2006; Wu et al., 2010). Microfluidic platforms are being used for formulating drug delivery carriers (Liu et al., 2017), their evaluation and screening of drug delivery systems (Björnmalm et al., 2014; Riahi et al., 2015; Chi et al., 2016; Damiati et al., 2018). Microfluidics-assisted drug screening involves investigating cell targeting, nanoparticle uptake, evaluating the efficiency of drug release and its effect on the host. The efficacy of such systems is predominantly dependent on the active targeting and ingestion of the drug loaded nanoparticle by the target cells cultured in the microenvironment. While the nanoparticle uptake is known to be reliant on nanoparticle material (Behzadi et al., 2017), size (Shang et al., 2014), surface chemistry (Albanese et al., 2012), charge (He et al., 2010), and perfusion flow rates (Jurney et al., 2017), the effect of microfluidic environment on the uptake efficiency is not yet explored. Specifically, it is not clear if cells cultured in micro environment ingest different amounts of nanoparticles than cells cultured in a macroenvironment (for example a petri dish). As microenvironment is known to alter the cellular behavior in terms of metabolism, growth (Paguirigan and Beebe, 2009), and the degree of endoplasmic reticulum stress (Su et al., 2013) it is logical to question if the microenvironment also influences nanoparticle uptake.

To systematically answer this question, we probe the effect of confinement on the cellular nanoparticle uptake via studying fluorescent nanodiamonds (FND) uptake in J774 murine macrophages. Deploying immune cells for drug delivery is an upcoming research interest (Anselmo and Mitragotri, 2014; Su et al., 2015; Wang et al., 2015; Zhang et al., 2018) and macrophages are being actively investigated for drug delivery applications (Gordon and Rabinowitz, 1989; Pei and Yeo, 2016). They have been used for drug delivery in cancer (Zhang et al., 2018), in inflammatory diseases (Ponzoni and Pastorino, 2018) and to the brain (Klyachko et al., 2017). In addition, the primary role of macrophages in biology is to engulf and clear the body of foreign material contamination as nanoparticles (Gustafson et al., 2015). Hence their innate high nanoparticle uptake efficiency can amplify and assert the difference in uptake efficiency if any, strictly due to the confinement of the cell surrounding. On the other hand, FNDs are emerging as a versatile tool for wide range of biological applications such as a biomarker (Fu et al., 2007), intracellular temperature (Sekiguchi et al., 2018), pH (Rendler et al., 2017; Fujiwara et al., 2019) and free radical sensor (Chipaux et al., 2018; van der Laan et al., 2018) for probing into the cellular metabolism. Moreover, owing to their tunable surface chemistry (Krueger and Lang, 2012) and photostable fluorescence (Schirhagl et al., 2014), FNDs have become an attractive drug delivery vehicle (Zhang et al., 2011; Ho et al., 2015). Their utility in cancer therapy (Liu et al., 2010; Xi et al., 2014), anti-HIV treatment (Roy et al., 2018) was recently reported. Therefore, macrophages and FNDs become a perfect model system for this work.

Here, we compare the FND uptake efficiency in J774 macrophages cultured in a petri dish and in a microfluidic channel. We show that the nanoparticle uptake in macrophages cultured in a petri dish is significantly higher than the uptake in cells cultured in a microfluidic chip. Moreover, we also show that the alteration in uptake efficiency is independent of the nanoparticle material through studying the uptake of fluorescent polystyrene particles in J774 cells. To investigate the underlying cause of this difference, we explored the effect of several parameters such as CO_2_ concentration in the medium, medium pH, availability of nutrients and the size and the material of the microfluidic chip. We further explore the general applicability of this observation in other cell types by investigating the FND uptake in Baby Hamster Kidney (BHK) -21 cells and the size and the material of the microfluidic chip.

## Materials and Methods

### Cell Culture Devices

As a representative of macroenvironment, we used sterile petri dish having four compartments (Greiner bio-one, Germany). As a representative of the microenvironment, commercially available (ibidi GmbH, Germany) microfluidic channels “C1” and “C2” (Schematics of the channels are shown in Figure 2C) which have similar lateral dimensions (50 mm length × 10 mm width) but 400 and 100 μm height respectively were used. In addition, we also used channels “C3” (ibidi GmbH, Germany) which were 400 μm tall but had smaller lateral dimensions of 17 mm length × 38 mm width compared to C1. All these devices were made out of biocompatible plastic type material (exact type of plastic unknown) and had a similar cover glass bottom. For one set of experiments, microfluidic devices made out of polydimetylsiloxane (PDMS) were used. This channel was purchased from BlackHole Lab. It had the same dimensions as “C1” and a cover glass bottom. All the devices were used directly without any further modifications.

### Fluorescent Nanodiamonds

In this work, we used commercially available FNDs having average size of 120 nm from Adámas Nanotechnologies, Inc. These particles are very well characterized in the literature. Although their average hydrodynamic diameter is 120 nm, their actual size varies between 50 nm and 200 nm (Adámas Nanotechnologies Inc, 2019). Furthermore, the size distribution of similar smaller FNDs have also been investigated in the literature (Hemelaar et al., 2017a). Moreover, crystallographic orientations of such particles and their shape is also characterized (Ong et al., 2017). These particles have oxygen terminated surface chemistry and zeta potential of their suspension in the DI water was observed to be ∼−20 mV (Hemelaar et al., 2017b).

### General Experimental and Imaging Protocol

For culturing J774 murine macrophages and HeLa cells, DMEM-HG complete medium comprising of DMEM-HG (Gibco), 1% Penn-strep (Gibco), 1% GlutaMax (Gibco) and 10% fetal bovine serum (Gibco) was used. On the other hand, RPMI medium consists of RPMI (Gibco), 1% Penn-strep (Gibco) and 10% fetal bovine serum (Gibco) was used for culturing BHK-21 cells. Experimental method was nearly same for all the experiments conducted with minor modifications to study the influence of the parameter of interest. All the experiments performed in this work comprise of the following basic steps: (a) seeding cells in a petri dish or a microfluidic device for a specific duration. Cell suspension concentration of 3 × 10^5^ cells/mL was used following the ibidi’s cell culture guidelines for microfluidic cell cultures. (b) FND incubation. To make FND suspension, 1.5 μL of 1 mg/mL 120 nm FND stock solution (Adámas Nanotechnologies, Raleigh, NC, United States) was added in 10% FBS which was subsequently added in 90% DMEM-HG or RPMI. (c) fixing the cells with 3.7% PFA and staining with DAPI and FITC-Phalloidin as previously demonstrated by Hemelaar et al. (2017b), (d) imaging the z-stack of fixed cells with laser scanning confocal microscope (Zeiss 780) and (e) quantifying the FNDs/cell with 3D object counter plugin of FIJI with threshold of 38 and 22 for Macrophages, BHK-21 and HeLa cells respectively and filter size from 2 to 30,000 which was constant throughout the quantification. For quantifying the polystyrene nanoparticles/cell, a threshold of 38 and filter sizes from 2 to 30,000 were used. We note that the parameters such as laser power, gain and magnification were maintained constant during the imaging. Table 1 gives the details of all the microscope setting used during the imaging. Table 2 gives the details of the experimental parameters used during different experiments. Baseline experiments conducted using J774, FNDs, and polystyrene nanoparticles are marked with yellow. For the rest of the experiments, parameters that retained constant or changed with respect to the baseline experiments are marked with green and red respectively.

**TABLE 1:** Details of the microscope parameters.

**Parameter**	**Details**
Microscope	LSM 780, AxioObserver
Objective	I LCI Plan-Neofluar 63x/1.3 Imm Korr DIC M27
Filters	-2147483648 – -2147483648
Excitations wavelengths used	408 nm (0.8%), 488 nm (6%), 561 nm (100%)
Detection wavelength	424 – 485 (detector gain: 518, detector digital gain: 1.2) 499 – 552 (detector gain: 647, detector digital gain: 1.6) 650 – 751 (detector gain: 810.3, detector digital gain: 1.0)
Image size	134.95 μm × 134.95 μm (512 × 512 pixels)

**TABLE 2:** Parameters used during different experiments.

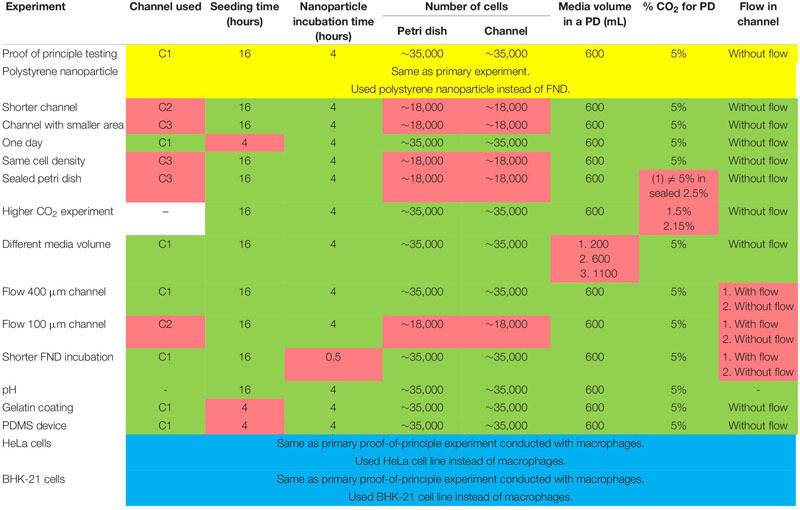

*Yellow color marks the baseline proof-of-principle experiments. Cells marked with green and red indicate the parameters retained constant and changed compared to the baseline experiment respectively.*

### Statistical Analysis

All the experiments done in this work “except PDMS experiment” were repeated at least 2–3 independent times. In every repetition, ∼50 cells were imaged per group. Using graphpad prism software, statistical tests were performed to test the statistical significance of the result. In particular, Mann–Whitney *U* test or Kruskal–Wallis test was conducted using GraphPad Prism version 8.0.0 for MacOS (GraphPad Software, San Diego, CA, United States^1^). Most of the data across different groups had non-normal distribution (as determined by D’Agostino and Pearson omnibus normality test and Shapiro–Wilk normality test of the GraphPad Prism version 8.0.0) and unequal number of measurements (in the range of 130 – 160). Therefore, non-parametric tests were used for determining the statistical significance. In the entire manuscript, statistical significance, if there is any, is indicated by ‘^∗^’. While determining the statistical significance of the results, we used *p* = 0.05. Specifically, if *p* was found to be less than 0.05 for the selected groups in the statistical test, then the difference in the test groups were determined to be statistically significant. In simple terms, *p* = 0.05 denotes that there is 5% chance that the test groups do not have significant difference in them although the statistical test characterizes them to be statistically different. Data is shown by box and whiskers graphs plotted using GraphPad Prism. Whiskers show 10–90 percentile.

### Overnight Perfusion Experiment Protocol

During the cell seeding, a 20 mL syringe (Terumo) containing fresh cell culture medium mounted on a syringe pump (NE-1000, Prosense B.V, Netherlands) was connected to the microfluidic channel via flexible silicon tubing and the luer lock connectors (ibidi GmbH, Germany). 1.5 h post cell seeding, the syringe pump was turned on to continuously pump the fresh medium through the channel at a rate of 0.3 and 0.1 mL/h for C1 and C2 channels respectively. During the experiment, microfluidic device was placed in the incubator (37°C, 5% CO_2_) where the syringe pump was positioned outside the incubator. Following the overnight incubation, syringe pump was disconnected and the FND incubations were conducted in the static conditions.

### pH Measurement

For measuring the cell culture medium pH, standard pH strips (mColorpHast pH Test Strips, MilliporeSigma, VWR) were used. Change in color of the test strip after adding a drop of medium on the test strip was visually compared with the calibration color grid provided by the manufacturer. The least count of measurement was 0.2–0.3.

### Cell Metabolic Activity Assay

Cells were seeded in the petri dish and the microfluidic channel where they were allowed to attach overnight without any perfusion. Post overnight incubation, the medium was discarded and cells were washed with sterile PBS. Next, 5 mg/mL MTT solution (sigma) was added over the cells. Culture devices were covered with aluminum foil to protect them from light and incubated at 37°C for 3 h. Then MTT solution was removed followed by the addition of the isopropanol over the cells to dilute the formazan produced by the cells. The isopropanol was removed from all the devices and placed in the 96 well plate to measure the absorbance 560 nm laser using the plate reader.

### Effect of Gelatin Coated Glass Bottom on the FND Uptake

First, 40 or 100 μL of 1% gelatin solution in water (Stock solution of 2% in H_2_O purchased from Sigma) were added to a petri dish and a channel respectively. Then the solution was allowed to dry for at least 2 h before washing the devices with medium to remove any unattached gelatin. Next, cells were seeded in the petri dish and the microfluidic channel and were allowed to attach for 4 h followed by 4 h of incubation with FNDs.

## Results and Discussion

### Comparing the Nanodiamond Uptake in Macrophages Cultured in a Petri Dish and in a Microfluidic Channel

**FIGURE 1:**
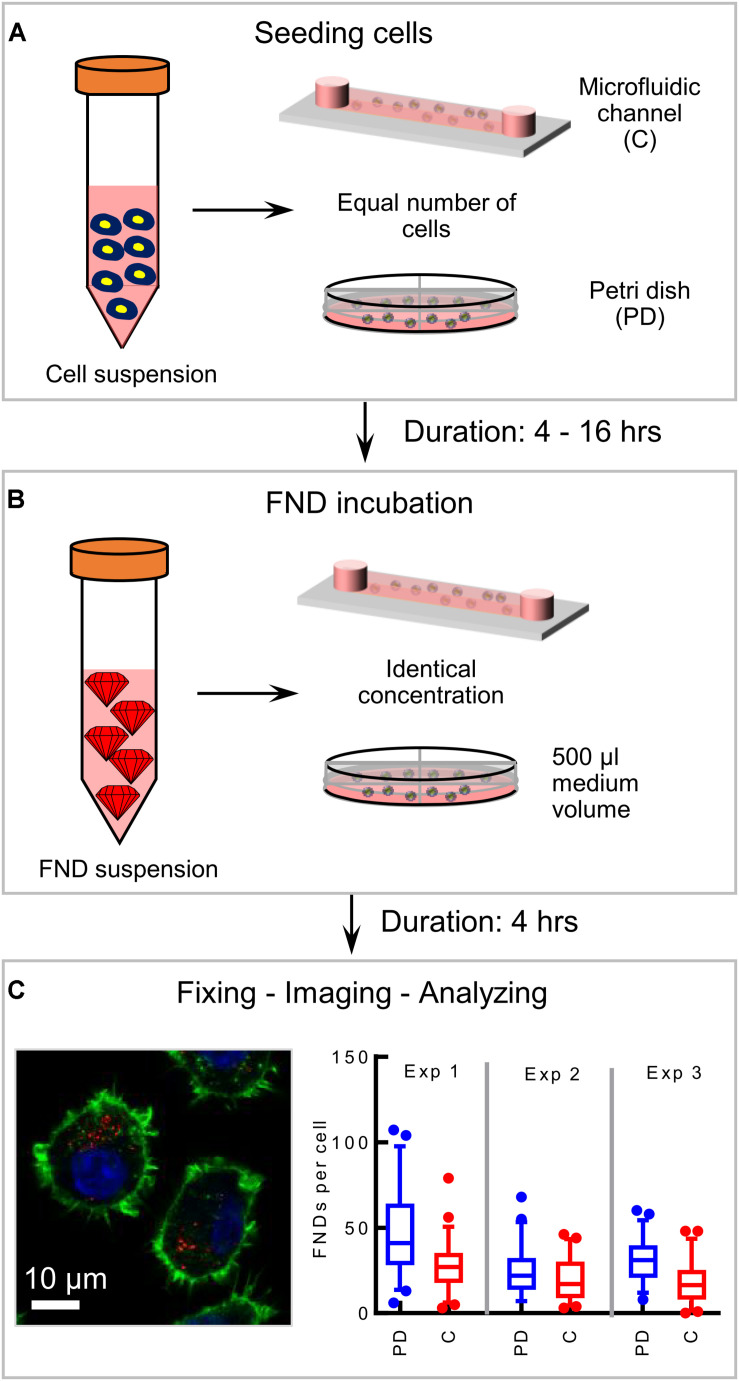
Schematic of the experimental methodology. **(A)** Seeding equal number of cells in the microfluidic device and the petri dish and allowing cells 4–16 h to attach to the glass bottom of the device. **(B)** FND suspension incubation for 4 h. **(C)** Fixed and stained macrophages are imaged in a laser scanning confocal microscope. Red dots in the image are FNDs where nucleus and cytoskeleton are indicated with blue and green color respectively. In every experiment, FNDs/cell are quantified in 50 cells per group. Each experiment is repeated three independent times.

In this work, we use commercially available 120 nm FND (Adámas Nanotechnologies, Inc., United States) and J774 murine macrophages (an immune cell line) as a model system to probe the effect of confinement on nanoparticle uptake. As a representative of a macroenvironment, we used plastic petri dishes having 4 quarters (Greiner bio-one, Germany) along with commercially available microfluidic devices with 0.4 or 0.1 mm channel height (ibidi GmbH, Germany). All the devices have same glass bottom. As shown in Figure 1, all the experiments performed in this work comprise of the following basic steps: (a) seeding cells and allowing them to attach to the glass bottom for a specific duration, (b) FND incubation, (c) fixing and staining cells as previously demonstrated37 followed by (e) imaging and quantification. Each experiment was repeated three independent times. In every experiment, 50 cells were imaged per group and the number of FNDs per cell was quantified using ImageJ. Hence 150 cells in total per group were analyzed across the three independent experiments as shown in Figure 1C. We note that, all the parameters used for imaging and analysis were maintained constant for all the devices.

Before investigating the nanoparticle uptake by cells, first we explored the interaction between the FNDs and the cell culture medium in the microenvironment. The interaction between the FNDs and medium may lead to aggregation which plays a crucial role in influencing the cellular uptake (Hemelaar et al., 2017b). To investigate, if this interaction is altered in the confined environment and if the confined environment promotes the FND aggregation, we checked the size of the FNDs incubated in the channel and in the petri dish using dynamic light scattering (DLS). Specifically, FND suspension alone (without cells) was placed in the petri dish and the channel. Then both the devices were maintained in the incubator for 4 h after which the suspension from the devices was collected with subsequent DLS characterization. Our results [given in the Supplementary Information (SI)] indicated no difference in the average hydrodynamic size of the nanoparticles collected from both the devices.

**FIGURE 2:**
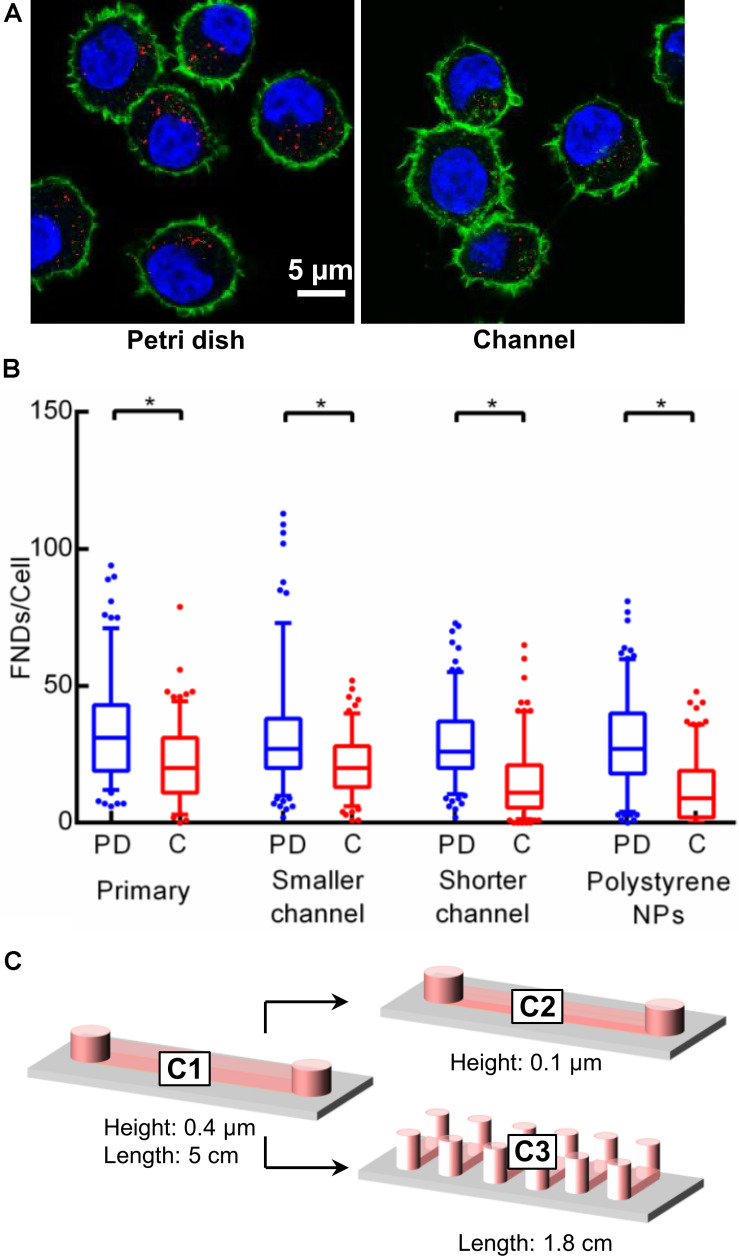
Results of the preliminary experiments. **(A)** Image of macrophages cultured in a petri dish and in a channel. Number of FNDs (red spots) taken up by the cell cultured in a petri dish is significantly higher than that in the channel. **(B)** Quantified results comparing the FNDs/cell in a petri dish against that in the channel for different experiments. In all the experiments, uptake efficiency in the petri dish was observed to be higher than in the channel. Mann–Whitney *U* test was used to check the statistical significance. **(C)** Size and shape of microfluidic channels used during the experiments.

To assess the effect of confinement on the cellular uptake, equal number of cells (∼2 × 10^4^) were seeded in one quarter of a petri dish and a 400 μm tall microfluidic device. Cells were then allowed to attach to the glass bottom while the devices were maintained in the incubator at 37°C and 5% CO_2_ overnight. Then the medium was removed and cells were incubated with freshly prepared medium containing 1.5 μg/mL FND for 4 h. Later, the FND suspension was removed from both devices followed by fixing, staining and imaging the cells to quantify the number of nanoparticles per cell. Figure 2A shows the representative images of the cells from both the devices acquired during this experiment, which were later used for the quantification. Figure 2B shows the results of the quantification. We found significantly higher uptake efficiency of macrophages cultured in a petri dishes than in a microfluidic device. To further validate and probe the observed effect of confinement on the nanoparticle uptake, experiments were conducted in devices having different channel geometries. In particular, in two independent sets of experiments, height and channel surface area was shortened to 100 μm (from 400 μm) “C2” and 0.6 cm^2^ (from 2.5 cm^2^) “C3” respectively. Schematics of the channel geometry are shown in Figure 2C. Higher uptake efficiency for cells cultured in the petri dish compared to microfluidic device was clearly evident. As nanoparticle material and shape are known to influence the cellular uptake, we studied if we see the same trends for different nanoparticle. Specifically, we explored the uptake of carboxyl terminated 100 nm polystyrene nanospheres in macrophages cultured in a petri dish and in a microfluidic device. These nanoparticles have similar surface termination and size as the FNDs. However, in contrast to FNDs with a flake like structure (Ong et al., 2017) these are spherical. Similar to FNDs, we found the polystyrene nanospheres uptake in cells from petri dish to be significantly higher than the cells in the microfluidic devices (Figure given in the Supplementary Information).

### Effect of Incubation Time and Cell Number on the Uptake Efficiency

**FIGURE 3:**
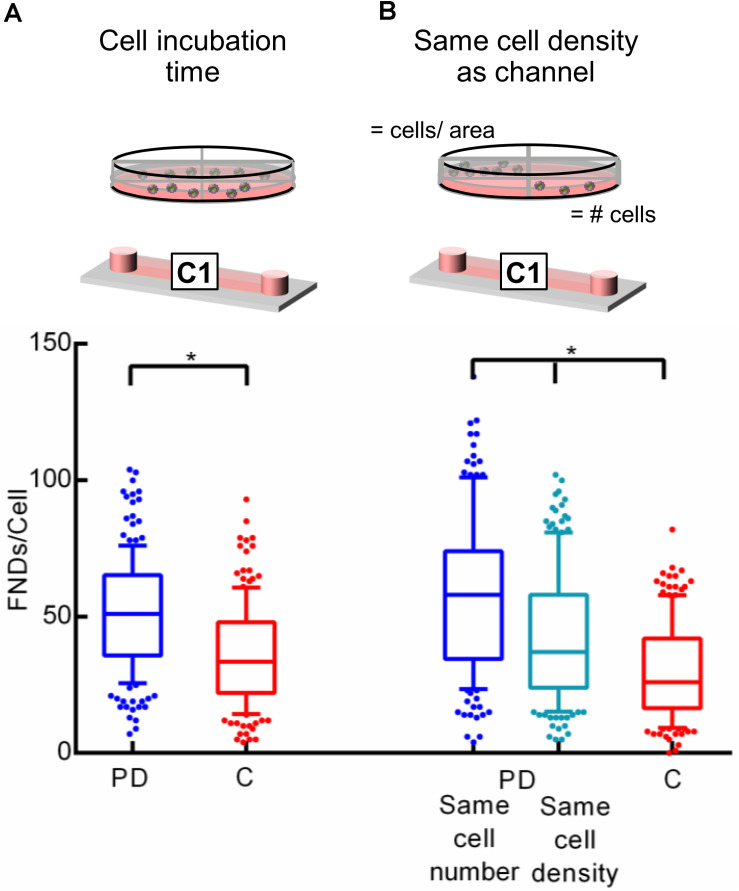
Investigating the effect of **(A)** cell seeding duration and **(B)** cell density on uptake efficiency. Statistical significance was calculated using Mann–Whitney *U* test and Kruskal–Wallis test in these experiments.

Figure 2 demonstrates experiments where we observed the significant difference in cellular uptake efficiency for both the devices. As elaborated above, cells were allowed to attach overnight during all the experiments. The population doubling time for J744 murine macrophages is ∼17 h [ATCC ATCC J774A.1 (ATCC^®^ TIB-67TM), 2018] which is less than the total duration of the entire experiment (∼20 h). Hence even though we seed the same number of cells in both devices at the beginning of the experiment, the number of cells in the petri dish may be a little higher compared to the channel. Therefore, to maintain the precise control over the number of cells, we limited the cell incubation time to 4 h followed by FND incubation of 4 h. Results are shown in Figure 3A where we see a similar trend in uptake efficiency as in the previous experiments. In another independent experiment, we maintained the same cell density in both the devices. It can be seen from the results in Figure 3B that the overall uptake in the petri dish quarter with the same cell density as in the channel is lower than the petri dish quarter with same cell number. This can be explained as the higher absolute number of cells led to larger distribution of FNDs among the cell population. However, this uptake was still significantly higher than the uptake in the channel.

### Effect of Cell Culture Media and Gaseous Environment on the Uptake Efficiency

**FIGURE 4:**
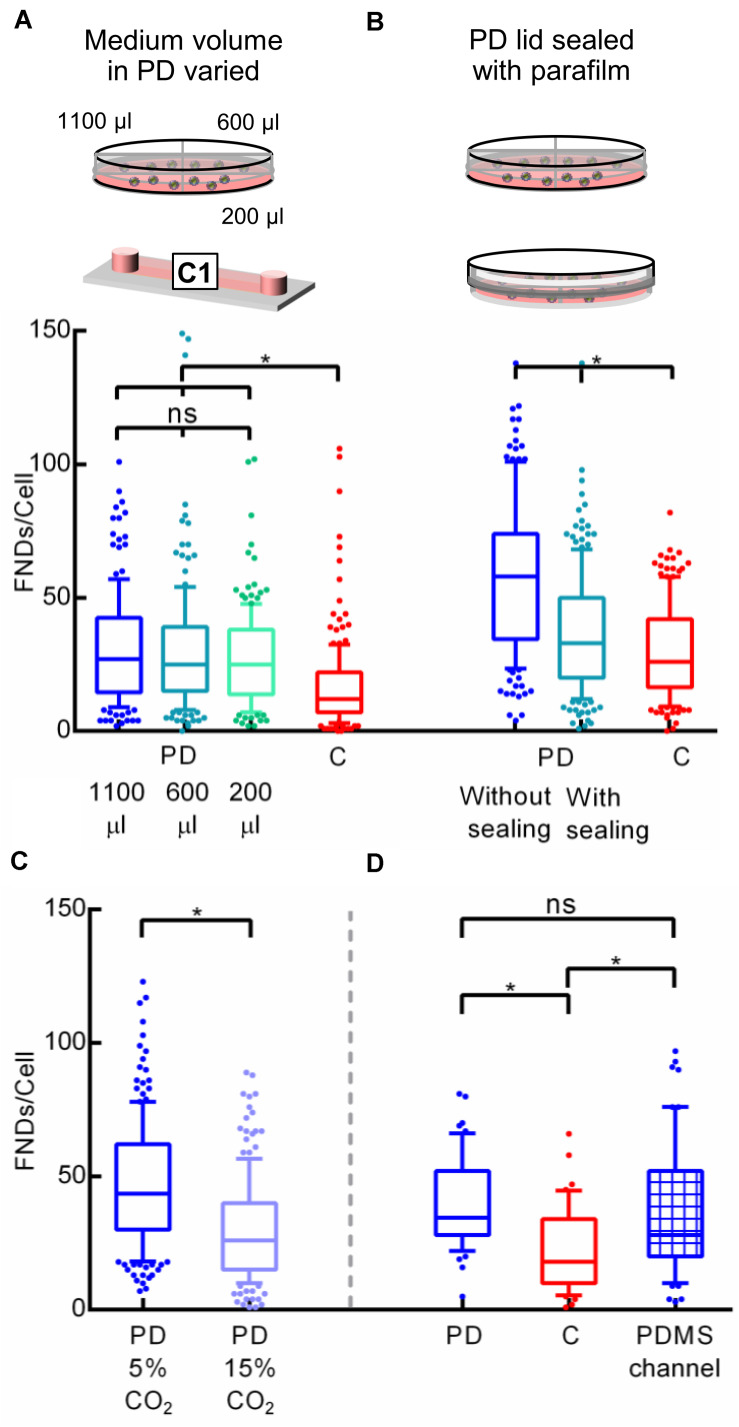
Investigating the effect of cell culture media volume and gas environment on the uptake efficiency. **(A)** Different media volume was placed in the petri dish during the overnight cell incubation. **(B)** Petri dish was sealed with the parafilm to alter the gaseous exchange between the petri dish and the incubator. **(C)** Difference in FND uptake in cells cultured in petri dishes placed in the incubator maintained at 5 and 15% CO_2_ overnight. **(D)** Comparing the uptake of cells cultured in the microfluidic device made out of PDMS against the uptake in cells cultured in the petri dish and channel made out of plastic.

To explain this difference in uptake efficiency between the two devices, gas environment and the limited volume of the cell culture medium appear to be the most obvious factors. It has been suggested that the cell medium needs to be changed after every ∼8 h on average in the microfluidic cell cultures as nutrient depletion and “waste” accumulation occurs at an elevated rate (Young and Beebe, 2010). This ensures the similar culture conditions as the macro environment. As phagocytosis is an energy dependent phenomenon, the reduced FND uptake in microenvironment could potentially be explained due to low availability and rapid depletion of the nutrients due to smaller cell culture medium volume. To investigate this further, the amount of cell culture medium in a petri dish during the overnight incubation was varied. Specifically, cells were seeded in three quarters of the petri dish containing 0.2, 0.6, and 1.1 mL cell culture medium. After incubating them overnight, they were exposed to equal amount of fresh FND suspension having identical concentration. From the results shown in Figure 4A, it is evident that the amount of medium in the petri dish or the concentration of nutrients and cellular waste from the medium do not have a significant impact on the uptake efficiency.

Similar to media volume, the gaseous environment in the microfluidic cell cultures is also considerably different compared to that in the petri dish. As the channels used during this work are made out of plastic, there is no free gaseous exchange between the medium over the cells and the gases in the incubator. Therefore, CO_2_ can buildup in the channel, which can alter the FND uptake. Hence to test the effect of the gaseous environment on the uptake efficiency, a petri dish was sealed using a parafilm after seeding cells. Then dishes/devices were transferred into an incubator for overnight incubation at 37°C and 5% CO_2_. After 16 h, parafilm was removed and the old medium was replaced with the fresh medium containing FND. Both the petri dishes without any sealing were maintained in the incubator at 37°C and 5% CO_2_. Uptake efficiency of cells cultured in the sealed petri dish was compared against the uptake efficiency of the cells cultured in a petri dish with loose lid and in a microfluidic device as shown in Figure 4B. Altering the gas environment through sealing the lid of the petri dish seemed to drastically reduces the cellular uptake efficiency. However, it was still significantly higher than what we observed in the microfluidic device. To further systematically test the effect of CO_2_ on FND uptake, uptake in cells cultured in the separate petri dishes maintained in the incubator at 37°C – 5% CO_2_ and 37°C – 15% CO_2_ overnight was compared. Following the overnight incubation, cells were given the fresh FND suspension and were maintained in the incubator at 37°C – 5% CO_2_ for 4 h. Results shown in Figure 4C corroborated well with our hypothesis about the important role of gaseous environment in altering the FND uptake as cells maintained in 15% CO_2_ ingested less particles. This experiment revealed an important result. Although both the petri dishes had fresh FND suspension and were kept in the same environment during the FND incubation, there was a great difference in the uptake efficiency. This can be connected only with the differences in the overnight incubation. Cells were likely stressed due to non-optimal CO_2_ concentration from which they could not recover quickly. This ultimately led to lower FND uptake.

Next, we compared the uptake in cells cultured in a petri dish and a microfluidic device made out of PDMS. Both channels had a cover glass bottom and all the dimensions of the PDMS channel were exactly same as channel “C1” described in section “Cell Culture Devices.” Among all the materials used for fabricating microfluidic devices such as polymers, glass, paper and plastics, PDMS is the most widely used material (Ren et al., 2013). Furthermore, PDMS devices allow gas exchange between the culture medium and the environment (which would be an incubator in our case). Thus, PDMS channels are a relevant alternative to test if the uptake is altered by the CO_2_ atmosphere in the channel during overnight incubation. In this experiment, an equal number of cells were seeded in the plastic “C1” channel, petri dish and a PDMS channel. Then cells were allowed to attach to the glass bottom for 4 h after which they were incubated with FND suspension for four more hours. All the incubations were carried out in the incubator maintained at 37°C and 5% CO_2_. After FND incubation, cells were washed, stained and imaged. Results of this experiment are shown in Figure 4D. It is very clear that uptake in the plastic channel was the lowest while the uptake in the petri dish and the PDMS channels were similar. Among the three devices used in this work, the petri dish and the PDMS channel allowed the gaseous exchange between the cells and the incubator where the plastic channel did not. Therefore, this experiment further bolstered our previous findings, which pointed toward the effect of non-optimal CO_2_ environment during the cell incubation on FND uptake.

### Overnight Perfusion

**FIGURE 5:**
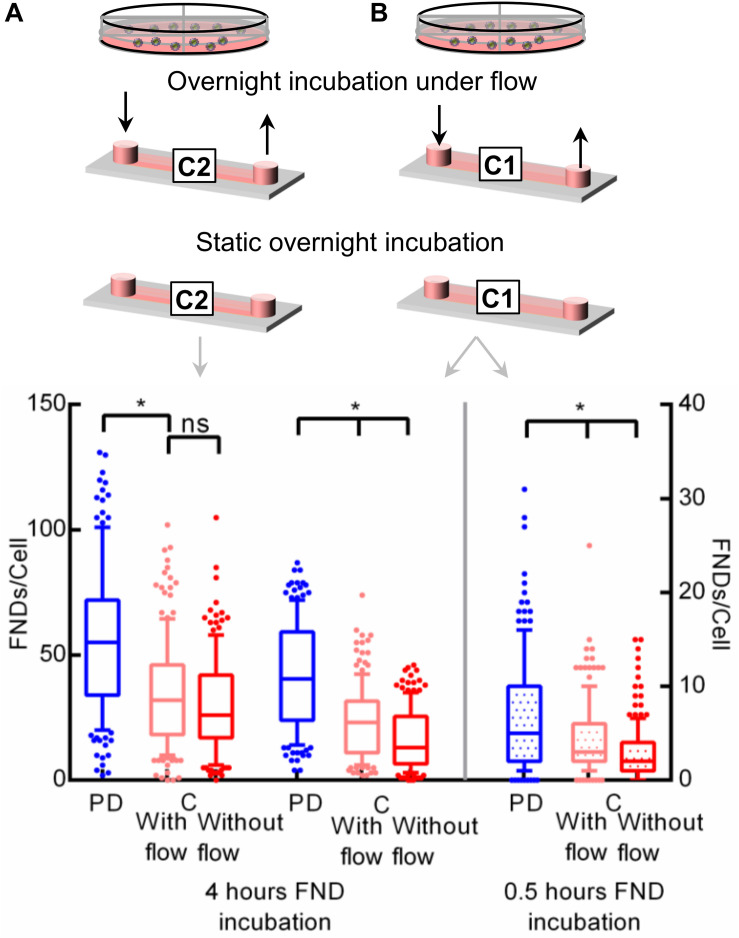
Investigating the effect of flow during the overnight incubation on the nanoparticle uptake. Two different channels with a height of **(A)** 400 μm or **(B)** 100 μm were used during this experiment.

To further confirm this observation that the gaseous environment during the overnight incubation affects the subsequent nanoparticle uptake efficiency, we connected the microfluidic device to an external syringe pump. After 1.5 h post seeding when cells were allowed to attach to the device substrate, the syringe pump was initiated to continuously pump the fresh medium through the device at a rate of 30 μL/h (400 μm tall, shear stress = 0.46 × 10^–3^ dyne/cm^2^) or 10 μL/h (100 μm tall, shear stress = 0.46 × 10^–3^ dyne/cm^2^) during the overnight incubation. This flow rate is higher than the ideal critical perfusion rate (CPR) for the straight microfluidic channel as described in the literature (Young and Beebe, 2010). CPR quantifies the frequency with which medium in the microfluidic device needs to be replaced compared to the static macro culture which given by CPR=LτR where *L* is the channel length and τ_*R*_ is the effective culture time which is typically between 8 and 12 h. This ensures continuous fresh medium supply to the cells without offering any significant shear stress on the cultured cells. Shear stress was calculated using the equations from the manufacturer’s technical specifications τ=η×104.7×Φ or τ=η×906.0×Φ for 400 and 100 μm tall channels respectively where τ,η,Φ are shear stress (dyne/cm^2^), dynamic viscosity (dyn.s/cm^2^) and flowrate (mL/min) respectively. In the literature, the effect of shear stress on nanoparticle uptake (Jurney et al., 2017), cytotoxicity (Rawat and Gadgil, 2016) and molecular delivery (Meacham et al., 2018) is very well established. However, the amount of shear stress in such investigations is typically on the order of 10–100 dyne/cm^2^, which is many orders of magnitude higher than the shear stress in this work. Hence effects of shear stress can be safely disregarded. After the overnight incubation, the syringe pump was disconnected from the device and cells were incubated with the FND suspension in a static environment. We compared the uptake efficiency of cells cultured in a petri dish, in a channel connected to a syringe pump and in channel without continuous supply of fresh medium as shown in Figure 5. In the case of channels with 100 μm height, higher cellular uptake was again observed in the petri dish. However, the uptake in both the channels was found to be very similar. In 400 μm tall channels with the overnight flow, cellular uptake efficiency was higher compared to the channel without flow. However, both channels had significantly lower uptake compared to the petri dish. Based on these results, we hypothesize the rapid CO_2_ buildup during the FND incubation itself. To test this hypothesis, we limited the FND incubation time to 0.5 h instead of 4 h. Quantified results which are shown in Figure 5 still show the same trend in uptake which we observed before. We would like to point out that we used static FND incubation in these experiments post overnight perfusion. Nanoparticle uptake during the perfusion incubation gets either enhanced or suppressed compared to the static incubation depending on the physical dimension and shape of the nanoparticle (Jurney et al., 2017). The commercially available nanodiamond suspension used in this work is far from homogeneous as far as the particle shape and size are concerned. Specifically, absolute particle size of 120 nm FND suspension (where 120 nm is the average hydrodynamic diameter determined by dynamic light scattering) can vary between 50–200 nm. Furthermore, the exact shape of these particles is also unknown. Previously, it was shown that FNDs with average hydrodynamic diameter of 25 nm have flake-like shape (Ong et al., 2017). However, similar investigations for larger particles have not been conducted. In addition, these particles are almost always prone to the minor aggregation in the cell culture medium, which will further alter their shape and size. Furthermore, the interaction between the FNDs and the tubing used for perfusion is also known. Therefore, we used static FND incubation alone in this work.

### Effect of Cell Culture Medium pH

Extracellular pH is also an indicator for dissolved CO_2_ or accumulated metabolic waste in the medium. Therefore, to further investigate our hypothesis of the CO_2_ accumulation, we measured the pH of the cell culture medium in all the devices post overnight and FND incubation. The pH of the medium in the microfluidic channel without flow post overnight incubation was found to be ∼4.7 whereas the pH in the petri dish and the channel with flow was found to be ∼8.0 and 8.3 respectively. The pH of the medium from the petri dish maintained in the 15% CO_2_ during the overnight incubation was ∼7.3 compared to the pH of ∼8.1 for the medium from the petri dish maintained in the 5% CO_2_. Thus, the groups having lower FND uptake across all the experiments had ‘relatively acidic’ medium during the overnight incubation. We note that, our pH measurement had a least count of 0.2–0.3 and it was based on manual observation of change in the color of the pH strips. Although all the devices were incubated with the fresh FND suspension following the overnight incubation, the pH of the FND suspension post 4 h incubation was found to be different in different devices. In particular, the pH was found to be ∼7.4 – 7.6 and ∼8.1 in the channels and in the petri dish respectively. Hence, it clearly highlights the buildup of the metabolic products or CO_2_ that occurs in 4 h of FND incubation. Moreover, the pH of the medium from both the channels (∼7.9) was found to be 2.5% lower compared to that of the petri dish (∼8.1) post the 0.5 h FND incubation. This demonstrates the accelerated buildup of the CO_2_ and cellular waste within 30 min post nanoparticle incubation. We point out that, the petri dish having the same cell density as the channel had higher pH (“relatively basic”) compared to the microenvironment post overnight incubation and post FND incubation. Thus, different cell number/density was not the dominant factor altering the medium pH.

**FIGURE 6:**
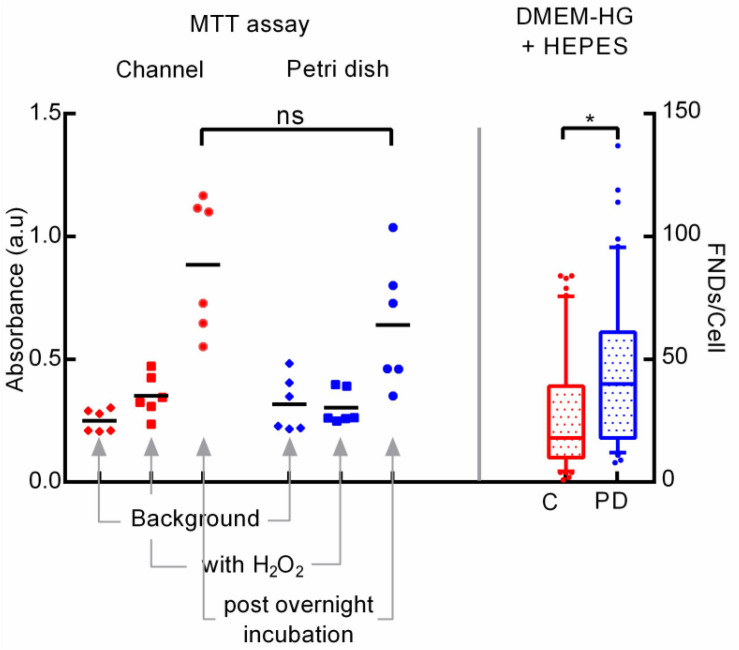
Exploring the cellular metabolic activity post overnight incubation through MTT assay **(left)** and the effect of adding HEPES buffering agent in cell culture medium on cellular uptake **(right)**.

Next, we tried to maintain constant pH across both the channel and the petri dish by adding 25 mM HEPES as a buffering agent in the DMEM-HG complete cell culture medium used for the experiments. After adding the HEPES, decrease in medium pH for channel post overnight incubation was limited to ∼6.1 rather than ∼4.7 as observed in the previous experiments without HEPES. Figure 6 (right) shows the difference in FND uptake.

Results from all these experiments clearly indicate the alteration in the medium pH leading to modifying the cellular activity ultimately resulting in the variation in cellular uptake. Hence, we further investigated the metabolic activity of the cells post overnight incubation using a colorimetric MTT assay which indicates the cellular metabolism. In this experiment, we probed the activity of cells cultured in both the devices post overnight incubation. Details of the experimental procedure are given in the materials section where Figure 6 (left) shows the results of the experiment. We found that, the average absorbance of 560 nm laser measured using the plate reader (Fluostar Optima) which corresponds to the amount of formazan and hence with the metabolic activity of the cells was higher in the microfluidic channel compared to the petri dish. Non-statistical significance as evaluated using the on-way ANOVA test between the two groups, despite of having considerably different average absorbance arise due to wider spread in the absorbance values. Absorbance from the negative control comprised of cells exposed to H_2_O_2_ which lowers the cellular metabolism was significantly lower compared to the test groups but slightly higher than the background absorbance by media alone without any MTT.

This results underlines the higher cellular metabolic activity of cells cultured in the microfluidic channel which corroborates well with the literature as Paguirigan and Beebe have demonstrated the similar results (Paguirigan and Beebe, 2009). Specifically, they reported the higher glucose consumption for mouse mammary fibroblast cells cultured in the microenvironment compared to the macroenvironment. Moreover, they also showed that cells in the microenvironment have higher metabolic rate in first 24 h compared to the cells in the macroenvironment. Here, authors used In Cells Westerns assay to quantify the activation of AMP activated protein kinase and S6 ribosomal protein which are signaling pathways associated with cell metabolism and growth. This result confirms the accelerated CO_2_ build-up due to higher cellular activity in the smaller medium volume which leads to the reduction in the extracellular pH which in turn modifies the FND uptake.

**FIGURE 7:**
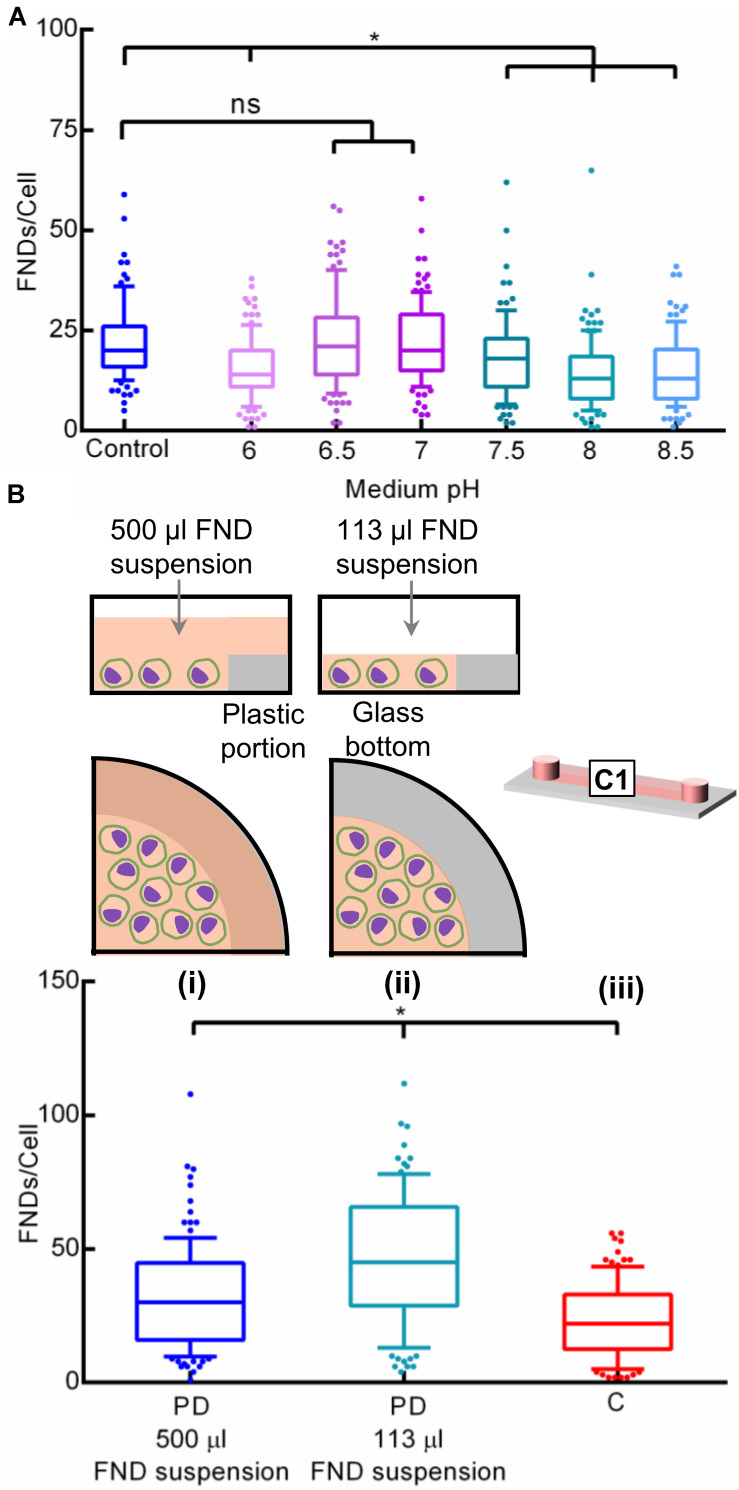
Investigating the effect of **(A)** pH of the cell culture medium in a petri dish and **(B)** surface area to volume (SAV) ratio on the nanoparticle uptake.

We further systematically evaluated the effect of medium pH on the FND uptake as extracellular pH is a well-established factor governing the cellular uptake of proteins and drugs (Friberg et al., 2003; Motizuki et al., 2004; Al-Khaza’leh et al., 2011). Here, we altered the pH of the cell culture medium from 6 through 8.5 via addition of hydrochloric acid or sodium hydroxide where the pH of the unmodified medium (control) was 7.8. Following the overnight incubation, old medium was discarded and all the devices were exposed to freshly prepared FND suspensions having the identical pH. Results of this experiment as shown in Figure 7A clearly demonstrate the effect of pH of the medium used during the overnight incubation on the cellular nanoparticle uptake. Therefore, there seems to be two crucial factors that affect the nanoparticle uptake in the microenvironment: (1) alteration in the cellular metabolism due to non-optimal CO_2_ environment or pH during the overnight incubation. (2) CO_2_ build up during the FND incubation itself.

### Surface Area to Volume Ration of the Cell Culture Device

Furthermore, surface area to volume (SAV) ratio is a critical factor in microfluidic cell cultures. Microfluidic platforms typically offer very high SAV ratios which results in significant dominance of surface-based phenomena such as nanoparticle uptake, gas diffusion, liquid evaporation, protein adsorption and efficient heat exchange (Wu et al., 2010). These critical factors can severely affect the microfluidic cell culture system, PDMS-based platforms in particular. However, as the commercial channels used in this work are made from plastic, we expect all the phenomenon except nanoparticle uptake to remain relatively insignificant. Hence, we assessed the influence of high SAV ratio of channel (22 cm^2^/mL) compared to the petri dish (3.8 cm^2^/ml) on unequal FND uptake. Specifically, we compared the FND uptake in a (i) petri dish containing 500 μL FND suspension, (ii) petri dish containing 113 μL FND suspension spread over shorter surface area and (iii) the channel as shown in Figure 7B. Device i contained ∼ 4.5 times higher numbers of FNDs compared to the petri dish ii. However, these FNDs were spread over the entire glass and plastic area of the petri dish. On the other hand, petri dish ii had a similar amount of FNDs as the microfluidic channel but they were spread over the smaller area compared to the channel given the surface area of the channel and the petri dish are 2.5 and 1.9 cm^2^ respectively. As evident from the results, FND uptake in the petri dish ii was found to be higher compared to petri dish i. Although device i had ∼ 4.5 times higher amount of FNDs available, it seemed to be irrelevant against the prevailing effect of surface area on the uptake efficiency. The influence of the SAV ratio on the cellular nanoparticle uptake is further supported by the higher uptake in the petri dish ii than the channel, both containing the same FNDs but prior had smaller surface area than latter.

Microfluidic environment not only offers the higher SAV, but it may also impart mechanical stresses and size alterations on the cells which may further modify the nanoparticle uptake. Wang and co-workers have demonstrated the size variations in macrophages probed using flow cytometry where they indicate that their sizes can vary significantly depending on the organ from which they were harvested (Wang et al., 2013). If the size of the cell is comparable to the microfluidic channel dimensions, then it can lead to cellular deformation and mechanical stresses in cells. In fact, microfluidic channels having characteristic dimensions (typically width or height of the channel) of < 30 μm are intentionally deployed to explore the influence of microenvironment on the modifications in cell polarity and shape (Terenna et al., 2008), cell motility (Faure-Andre et al., 2008), disease pathophysiology (Higgins et al., 2007), cancer metastasis (Chaw et al., 2007), fundamental cell biology (Minc et al., 2009) or bacterial cell shape (Takeuchi et al., 2005). In many of these studies, microfabrication feature size as small as 4 μm was even used.

Here, to assess the presence of shape deformation caused by the microenvironment, we acquired 3-D image stack of cells cultured in different culture devices using laser scanning confocal microscope with constant slice thickness of 1 μm. Z-stack images reveal that the cells cultured in the 100 μm and 400 μm tall microfluidic chips and the petri dish have the comparable average cell height of ∼15 μm. This result highlights that the height of the cells is not only completely independent of the culturing device but moreover it is about < 20% compared to the 100 μm channel height. Therefore, the possibility of cell shape deformation and mechanical stresses caused by the microenvironmental confinement leading alterations in nanoparticle uptake could be eliminated.

### Comparing the Nanodiamond Uptake in BHK-21 and HeLa Cells Cultured in a Petri Dish and in a Microfluidic Channel

**FIGURE 8:**
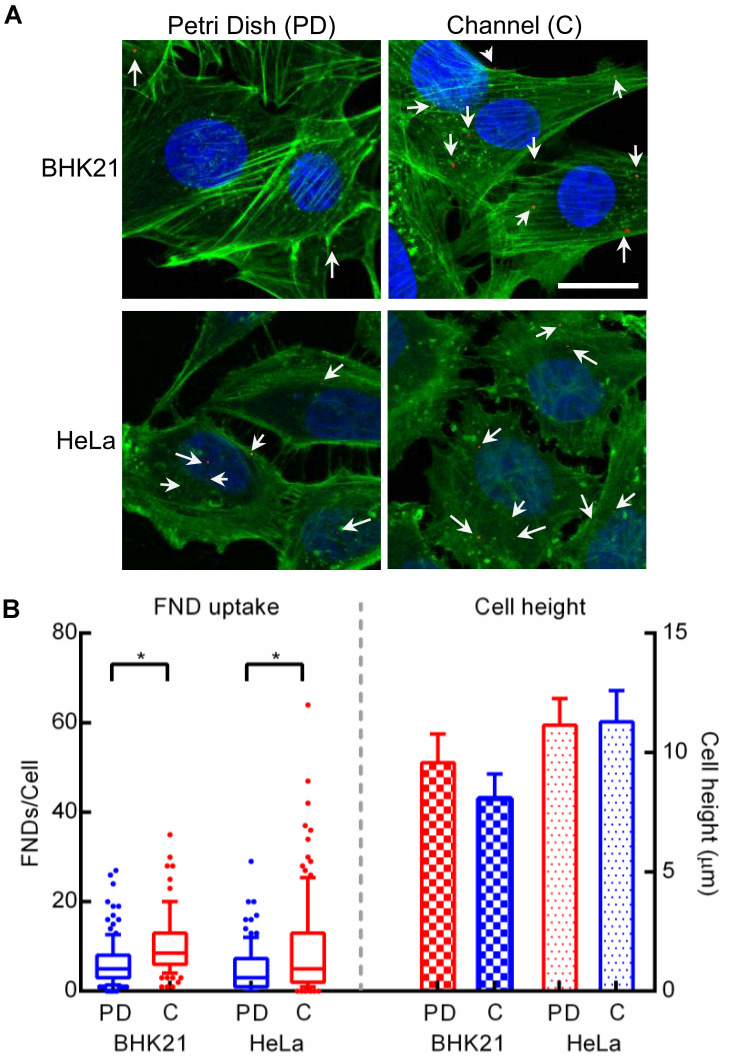
**(A)** Confocal microscopy images of BHK-21 and HeLa cells cultured in a petri dish and a microfluidic channel. Scale bar indicates 20 μm. **(B)** FND uptake (left portion) and cell height (right portion) observed in these two cell types across both the devices.

To further explore the general applicability of these results to other cell types, we investigated the FND uptake in BHK-21 cells cultured in a petri dish and in a microfluidic channel. As shown in Figure 8, the uptake of cells in a petri dish was found to be lower compared to that of cells in a microchannel. This observation is completely contradictory to our findings with macrophages. This observed contradiction can be explained by differences in glucose/energy requirement/metabolic rates of macrophages and BHK-21 cells and the different cell culture media used for these cells. Specifically, for culturing macrophages, DMEM- high glucose with GlutaMax supplement was used where for BHK-21 cells were cultured in RPMI medium containing HEPES buffering agent without any additional supplements. One more prominent difference found for these two cell types was the medium pH post overnight and FND incubation. In particular, medium pH post overnight incubation and post FND incubation was found to be identical for all the devices for BHK-21 cells. This is different from what we found for macrophages. As shown earlier by the experiments with macrophages, SAV ratio is a key factor directing the nanoparticle uptake. Although the devices hence the SAV ratio used for the experiments with J774 and BHK-21 were identical, there is a prominent difference in the morphology and the surface area of the BHK-21 and J774 cells. In particular, J774 having circular cross section had the average surface area of ∼420 μm^2^ compared to the BHK-21 cells having wide-spread morphology having ∼2400 μm^2^ surface area. Therefore BHK-21 cells had better surface coverage and thus had “access” to larger amount of nanodiamonds.

As microfluidic device technology is a promising tool for cancer diagnosis and cancer biology (Zhang and Nagrath, 2013), we also investigated the applicability of our results by exploring the nanodiamond uptake in HeLa cells, the most commonly used cancer cell line. Moreover, morphology of HeLa cells is similar to that of BHK-21 cells. This allowed us to verify if the uptake in the BHK-21 cells is indeed influenced by larger surface coverage due to widely spread morphology. In the case of HeLa cells, the results shown in Figure 8B were found to be similar to results for BHK-21 cells where uptake in cells cultured in the microfluidic devices was more compared to cells in a petri dish. In addition, we observed the distribution in FND uptake across HeLa cells to be wider than for BHK-21 or macrophages. It can also be noted that, although the difference in uptake across the two devices was statistically significant in HeLa cells, it was less prominent than the difference observed in BHK-21 cells and macrophages. This is likely due to the overall lower average uptake in HeLa cells than BHK-21 cells. In fact, among the three cell types studied in this work, uptake in HeLa cells was least. This result is also consistent with observations from other experiments in our group. Although the morphology of BHK-21 and HeLa cells is similar, there is a considerable difference in their height and surface area, which was quantified using microscopic images. The result shown in Figure 8B, shows that BHK-21 cells are flatter than HeLa cells and have a higher surface area (∼2400 μm^2^) compared to HeLa cells (∼1700 μm^2^). This result confirms the influence of cell shape on the nanodiamond uptake.

### Effect of Gelatine Coating on FND Uptake

**FIGURE 9:**
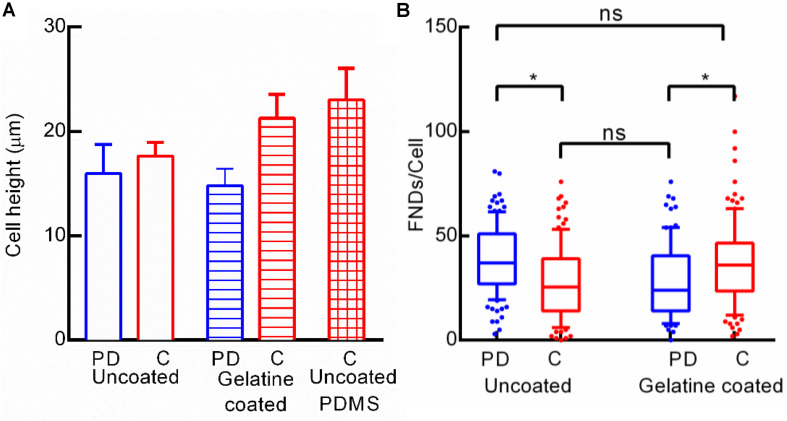
**(A)** Height of macrophages cultured in uncoated and gelatin coated devices and uncoated microfluidic channel made out of PDMS. **(B)** Effect of gelatin coating the glass bottom of the channel on FND uptake.

As the cell shape seemed to be a critical factor in nanoparticle uptake in the case of BHK-21 and HeLa cells, we also explored if the difference in the cell shape (mostly height as round shaped surface area is very similar) of macrophages cultured in a petri dish and microfluidic channel is altering the FND uptake. Therefore, we quantified the height of ∼20+ random cells manually from z-stack images recorded using a confocal microscope. As shown in Figure 9A, the height of the cells from both the devices was very similar but still there was a significant difference in FND uptake across those two devices which can be clearly seen in Figure 9B. To further explore this hypothesis, we coated the glass bottom of both devices with 1% gelatin in water solution which would promote the cellular adhesion and spreading on the surface. This would lead to the modification in the FND uptake. The height of the cells in gelatin coated channel was found to be considerably higher than that in gelatin coated petri dish which was similar to uncoated device. However, the FND uptake in cells cultured in gelatin coated channel was higher than that of gelatin coated petri dish. This trend is exactly opposite of the uncoated device. As demonstrated earlier in Figure 4D, uptake in uncoated PDMS channel was similar to uptake in uncoated petri dish although the height of cells cultured in the uncoated PDMS device was found to be highest. Therefore, we could not find any clear connection between, gelatin coating, cell height and uptake in the case of macrophages.

## Conclusion

In summary, we confirm the impact of the microenvironment on the nanoparticle uptake compared to the macroenvironment. Microenvironment primarily modifies the CO_2_ environment and the medium pH in addition to offering a very high SAV ratio. All these factors lead to the alteration in the uptake efficiency of cells cultured in the microenvironment compared to the macroenvironment. Although the extracellular pH is a known factor to modify the cellular uptake of proteins and drugs (Friberg et al., 2003; Motizuki et al., 2004; Al-Khaza’leh et al., 2011), the exact cascade signaling/reaction mechanism that connects the non-optimal extracellular pH or CO_2_ environment to intracellular environment to the alteration in the FND uptake is still unknown for this particular study. In this study, we pointed out the factors responsible for change in uptake in the microfluidic environment by carefully designing the experiments. In addition, we also found that the manifestation of the effect of microenvironment on the nanoparticle uptake is not similar across all the cell types. This work also highlights the effect of cell morphology and the available cell surface area on the uptake efficiency. Specifically, for cells such as HeLa and BHK-21, which have more spread morphology, their surface area and the cell height dominate the nanoparticle uptake compared to other factors. We believe that the findings from this work could help improve the design of the microfluidic platforms and tailor the pH and gas environment recipes to trigger or prevent the uptake rates depending on the specific cell type. Additionally, it is important to be aware of such differences and understand their causes when working with microfluidic systems for nanoparticle uptake.

## Data Availability Statement

All datasets generated for this study are included in the article/Supplementary Material.

## Author Contributions

VD came up with the hypothesis for this work, designed and coordinated all the experiments. VD and LN conducted the preliminary proof-of-principle experiments. RSh carried out the bulk of the experiments and image analysis. AM carried out the MTT assay. VD and RSc wrote the manuscript. All the authors contributed to the article and approved the submitted version.

## Conflict of Interest

The authors declare that the research was conducted in the absence of any commercial or financial relationships that could be construed as a potential conflict of interest.

## Footnotes

1 www.graphpad.com
