# Supplementary material for: Micro Versus Macro – The Effect of Environmental Confinement on Cellular Nanoparticle Uptake
Source: Front Bioeng Biotechnol. 2020 Jul 24;8:869. doi: 10.3389/fbioe.2020.00869 (PMC7393206; doi:10.3389/fbioe.2020.00869)
Supplement: Supplementary file 1 [file Presentation_1.pdf]

Supplementary Material

1 Quantification of the hydrodynamic diameter of the FND suspension before and after incubation in the culture device

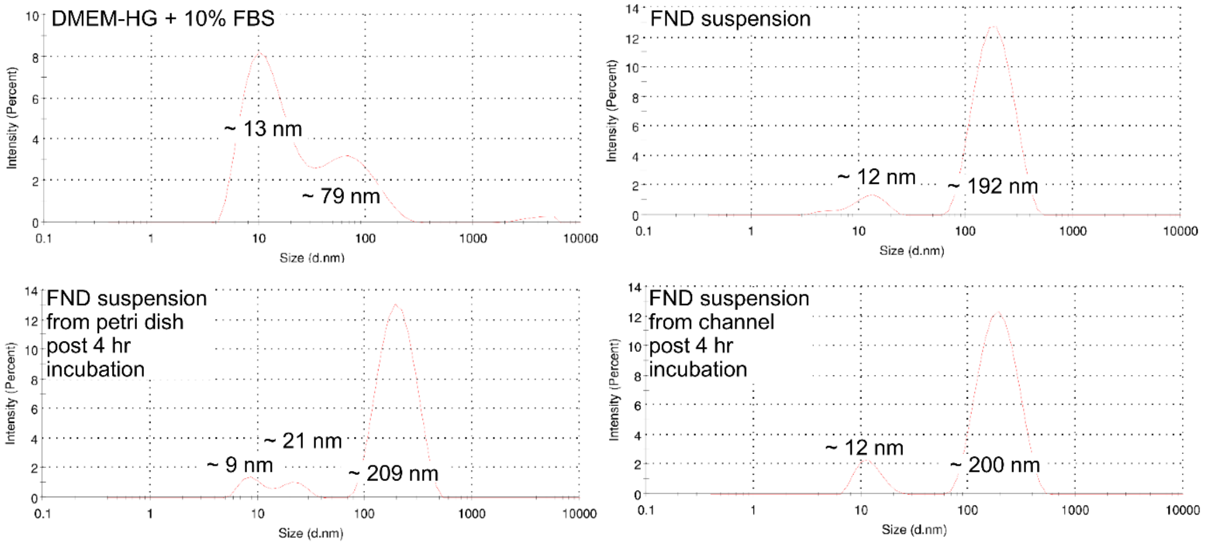

Supplementary Figure 1. hydrodynamic diameter of the FND suspension determined using dynamic light scattering before and after incubation in the culture device.

2 Uptake of polystyrene nanoparticles in J774 macrophages

Prior to using polystyrene nanoparticles, the commercially available suspension was diluted with MQ water to obtain 1 mg/ml stock solution. This stock solution was mixed with FBS and then with DMEM-HG to prepare the nanoparticle suspension. Size of the nanoparticle or aggregates if any was checked using dynamic light scattering (Malvern). We found the size of the FND and polystyrene to be comparable in the nanoparticle suspension.

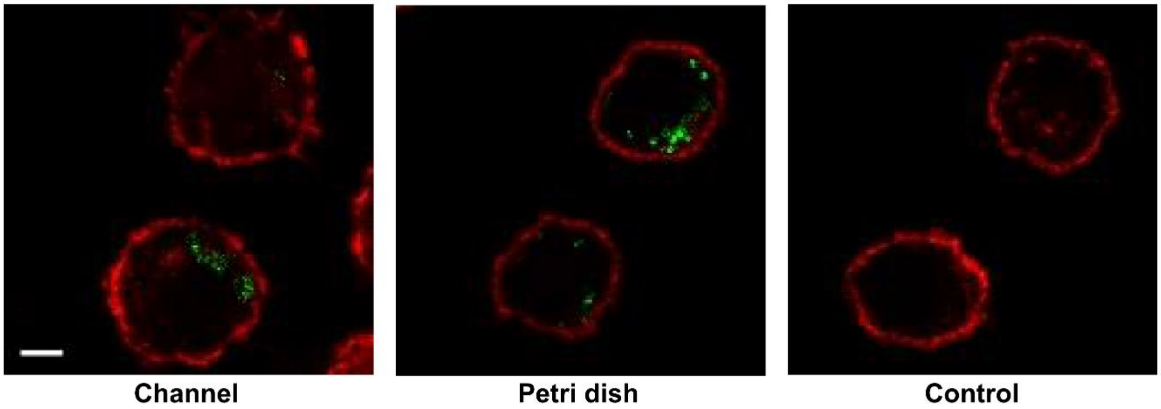

Supplementary Figure 2. Cellular cytoskeleton shown in red color in the images above is stained with TRITC-phalloidin. Scale bar indicates 5 µm.
